# Spawning and larval development of two tropical cowries (Gastropoda: Cypraeidae), *Cypraea tigris* and *Mauritia arabica* under laboratory conditions

**DOI:** 10.1098/rsos.221332

**Published:** 2023-04-12

**Authors:** Teresa Stephanie Tay, Hsien Rong Samuel Lee, Mei Lin Neo

**Affiliations:** Tropical Marine Science Institute, National University of Singapore, 119277, Singapore

**Keywords:** cowry, Cypraeinae, captivity, microalgal diet, mariculture, reproduction

## Abstract

The spawning and larval culture of cowrie (family Cypraeidae) are both difficult and little known, in part due to the long planktonic period of most species. In this study, we describe the captive spawning behaviour and larval development of two tropical cowrie species, *Cypraea tigris* and *Mauritia arabica*. Both species brooded over their egg masses before hatching occurred and larvae were collected for culture under laboratory conditions. The brooding period for *C. tigris* was between 7 and 17 days, and freshly hatched veligers were approximately 200–240 µm in size. *Cypraea tigris* larvae were reared for up to 37 days in culture but did not achieve successful settlement. The brooding period for *M. arabica* was between 7 and 10 days, and hatched veligers were approximately 160–205 µm in size. The first settled juvenile *M. arabica* was observed at 70 days post-hatch. Our findings from this study represent the first comprehensive documentation of successful metamorphosis of Cypraeidae larvae, particularly *M. arabica*, into early-stage juvenile.

## 1. Introduction

Cowries are a group of marine gastropods under the family Cypraeidae characterized by their smooth, oval-shaped shells and extendable mantle [1,2]. In the fifteenth and sixteenth centuries, cowries were heavily harvested by coastal islanders as food [3,4], and their shells were used for the handicraft industry [5,6] or as currency [7,8]. In recent times, the cowries, especially the rare species, have become popular among shell collectors and aquarium hobbyists because of their polished ornate shells and striking appearances when their mantle is fully extended [6]. Herbivorous species such as the *Mauritia arabica* and *Erronea errones* are also often favoured by aquarium hobbyists as algae controls since they consume algae voraciously. The published research on the Cypraeidae family has largely focused on the taxonomy, systematics and biology of species (e.g. [1,9–11]), with fewer studies focused on their reproduction, spawning and larval life cycle (e.g. [12,13]). Notably, studies on cowrie larvae have not reported success in closing the life cycles, which is a critical part in managing species numbers and conservation.

**Figure 1.**
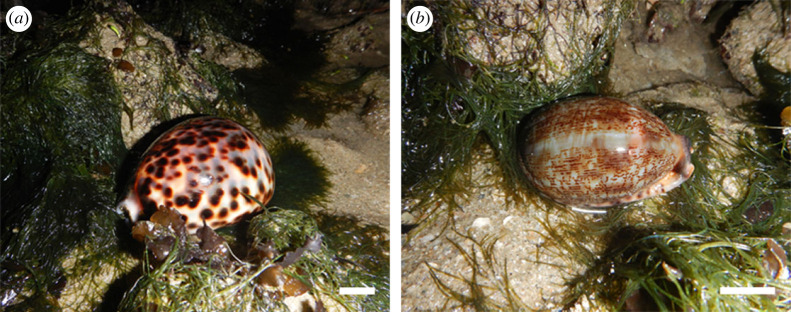
Field sightings of (*a*) *Cypraea tigris* (tiger cowrie) and (*b*) *Mauritia arabica* (Arabian cowrie) in Singapore. Scale bar: 2 cm.

This study focused on two tropical cowrie species: *Cypraea tigris* (tiger cowrie) and *Mauritia arabica* (Arabian cowrie) (figure 1). *Cypraea* *tigris* is one of the largest cowrie species, where an adult can measure up to 15 cm in length [14]. Although its common name relates to a ‘tiger’, the shell is spotted and not striped. It is widely distributed across the Indo-Pacific region, from the eastern coast of Africa to Micronesia and Polynesia, the Coral Sea and Philippines. This species is generally found between depths of 10 and 40 m, and often associated with *Acropora* corals [15]. While most cowries are nocturnal, spending much of their time hidden under rocks or dead corals, the large and robust *C. tigris* have also been observed to graze freely during the day [2]. Juvenile *C*. *tigris* are herbivorous and feed on algae, while the carnivorous adults feed on sponges and corals [16,17]. Once a common sight on the reefs, the numbers of *C*. *tigris* are declining due to shell collection and destruction of habitats [18,19]. The *M*. *arabica* is a medium-sized cowrie, which can reach up to 8 cm in length [20]. It has a widespread distribution in the Indo-West Pacific, from East and South Africa (but not Red Sea or Persian Gulf) to the eastern Polynesia [21]. This species is usually found in low intertidal zones to shallow depths of less than 10 m. In contrast with *C*. *tigris*, the *M*. *arabica* dwells under boulders and rocks, increasing activity at night in search for food. This species is an omnivore feeding on algae and small invertebrates [16]. Both the *C*. *tigris* and *M*. *arabica* are endangered in Singapore because of habitat loss and over-collection [15].

Members of the family Cypraeidae are largely gonochoric, with internal fertilization taking place between copulating pairs [22]. Most species display internal embryonic brooding as a form of maternal protection, where the female will deposit egg capsules and cover the egg mass with its foot prior to hatching of larvae [13,23,24]. This brooding behaviour has been suggested to be an adaptation to increase reproductive success in gastropods [25,26]. The tropical cowries typically have a high fecundity, with shorter brooding periods (also known as encapsulation periods) of up to three weeks and produce numerous planktotrophic veligers per capsule [27,28]. By contrast, several temperate cowrie species of genera *Zoila*, *Notocypraea* and *Austrocypraea* generally have low fecundity, with long brooding periods (i.e. 40–55 days) and direct development to one juvenile cowrie per capsule [12,29].

To date, most reproduction studies on cowries have not reported success in rearing larvae to settlement in captivity. These studies generally describe the morphological observations of egg masses and their capsules collected from the wild, as well as the early larval development (e.g. [27,28,30]). While some of these studies had tried incubating egg masses to hatch larvae for culture under laboratory conditions, these larvae rarely lasted more than a week (e.g. [27,31,32]). Only two studies showed moderate success in rearing older Cypraeidae larvae: Renaud [22] had cultured *Monetaria moneta* larvae for 38 days post-hatching (dph), while Jagadis *et al*. [33] had collected larvae from an egg mass of a *Cypraea tigris* in captivity and maintained the larvae cultures up to 16 dph. Both studies, however, only sparsely described the early larval development and no larval settlement was observed. To our best knowledge, no studies have yet to extensively describe the process of larval development to settlement, or the larval shell anatomical morphology of any Cypraeidae species. In this study, a breakthrough in the reproduction of two tropical cowrie species (*Cypraea tigris* and *Mauritia arabica*) in captivity is reported, which details the spawning, brooding and larval development processes under laboratory conditions.

## 2. Material and methods

### 2.1. General broodstock maintenance

Broodstock of both cowrie species (*C*. *tigris*: *n* = 14, *M*. *arabica*: *n* = 7) were separately housed in two 288 l tanks (80 × 80 × 45 cm; L × W × H) in an outdoor aquarium. The seawater system in these tanks was flow-through, sand-filtered seawater (FSW) directly from the sea, with an average water temperature of 30°C (± s.d. 1°C) and salinity of 31 practical salinity unit (psu) (± s.d. 1 psu). The *C. tigris* broodstock consisted of individuals purchased from the aquarium trade (imported from the Philippines: *n* = 9 and Indonesia: *n* = 1) and collected locally from three sites in Singapore (Pulau Semakau: *n* = 1, Terumbu Raya: *n* = 1 and Tanjong Rimau: *n* = 2), while the *M*. *arabica* were all collected locally (St John's Island, Lazarus Island and East Coast Park). The relevant permits (e.g. export and import permits, research permits) were secured prior to the purchase and collection of broodstock of the respective species. All individuals were photographed, catalogued and given a unique identifier for monitoring and tracking purposes. A fixed diet was provided to the broodstock three times a week: individuals of *C. tigris* were fed 6–8 g of shelled prawn and 0.03 g of cucumber, while individuals of *M. arabica* were fed 3 g of shelled prawn and 0.05 g of cucumber. General maintenance, such as cleaning the tanks to remove faecal matter and clearing excess foods to prevent fouling of the water were regularly carried out. The wet weights of the animals were measured every two months to ensure that they were in healthy condition.

### 2.2. Brooding and spawning

Cowries were regularly checked between July 2021 and September 2022 for brooding behaviour. Observations of clumping were also recorded. When a brooding cowrie with an egg mass was identified, the individual would be isolated from the other resident cowries for further observations. Throughout the egg mass brooding period, the female cowrie was mostly left undisturbed except for brief removal by gently prying its foot to photograph egg mass and collect capsules for measurements. For both species, where observations were possible, the following measurements were taken: (i) the length and width of egg mass (*N* = 16 for *C*. *tigris* and *N* = 5 for *M*. *arabica*), (ii) the length and width of capsules (*n* = 5 per egg mass; *N* = 15 for *C*. *tigris* and *N* = 5 for *M*. *arabica*), and (iii) the average number of embryos per capsule (*n* = 3 per egg mass; *N* = 15 for *C*. *tigris* and *N* = 5 for *M*. *arabica*) was estimated by breaking open the capsule and counting all the encapsulated embryos present. The total number of larvae produced by each brooding individual was also estimated by counting the hatched veligers. Short notes on embryogenesis were recorded, where observations could be made.

### 2.3. Larval culture and development

As previous cowrie larval cultures have not been successful in obtaining settled juveniles, the culture protocol used here is a modification from previous experiences with molluscan larval cultures with the goal to achieve juvenile settlement [34]. The hatched veliger larvae were hand-collected by filtering through a 100 µm mesh and reared in 1.8 l glass beakers containing 50 µm FSW at a density of 0.5 larvae ml^−1^. Cultures were reared in the controlled environmental chambers at 28°C, provided with moderate aeration initially that increased from 5 dph, and followed a 12 : 12 light : dark cycle. Maintenance of cultures was carried out three times per week, starting with a 50% water change, followed by feeding a diet of *Tisochrysis lutea* microalgae. The concentrations of microalgae varied according to the age of larvae, ranging between 5000 and 20 000 cells ml^−1^ day^−1^ (table 1). Larvae were reared until competent for settlement, and the settled juveniles were transferred into 100 ml glass dishes with tiles conditioned in flow-through aquarium. Competent larvae were determined based on the following characteristics: tentacles of equal length, partially reabsorbed velum and signs of crawling.

**Table 1.** Concentrations of *Tisochrysis lutea* fed to *C. tigris* and *M. arabica* larvae over culture period.

days post-hatching (dph)	no. of cells ml^−1^ day^−1^
0–3	5000
4–8	10 000
9–13	15 000
>14	20 000

To capture the various larval developmental stages, a random sample of live larvae (*n* = 15) was extracted once every 2–3 days and observed under a microscope to examine their morphological changes throughout the rearing period. To guide our observations, the terminology of larvae and juveniles followed Knight *et al.* [35]. A separate random sample of live larvae (*n* = 15) was retrieved from each egg mass batch of respective cowrie species for shell length measurements at regular intervals up to 18 days and 21 days for *C*. *tigris* and *M*. *arabica*, respectively. Larval survivorship of each egg mass batch was determined by enumerating live/dead larvae from two 1 ml aliquot samples obtained at random after concentrating the larvae during washing. The mean larval survival proportions for up to the first 11 days were averaged across five and four batches of *C*. *tigris* and *M*. *arabica*, respectively. Post-settlement survival of juveniles was also noted, where available.

### 2.4. Anatomy of cowrie shells

Cowrie larvae shells were collected at various development stages and fixed in absolute ethanol for scanning electron microscopy (SEM). After fixation, the shells were processed by washing in 6% sodium hypochlorite and followed by rinsing multiple times with distilled water. The processed shell samples were then mounted onto copper tape and left to air dry for 24 h, after which they were sputter-coated with platinum and examined under a SEM (Model: JSM-6010PLUS).

## 3. Results

### 3.1. *Cypraea tigris* (tiger cowrie)

#### 3.1.1. Brooding and spawning

**Figure 2.**
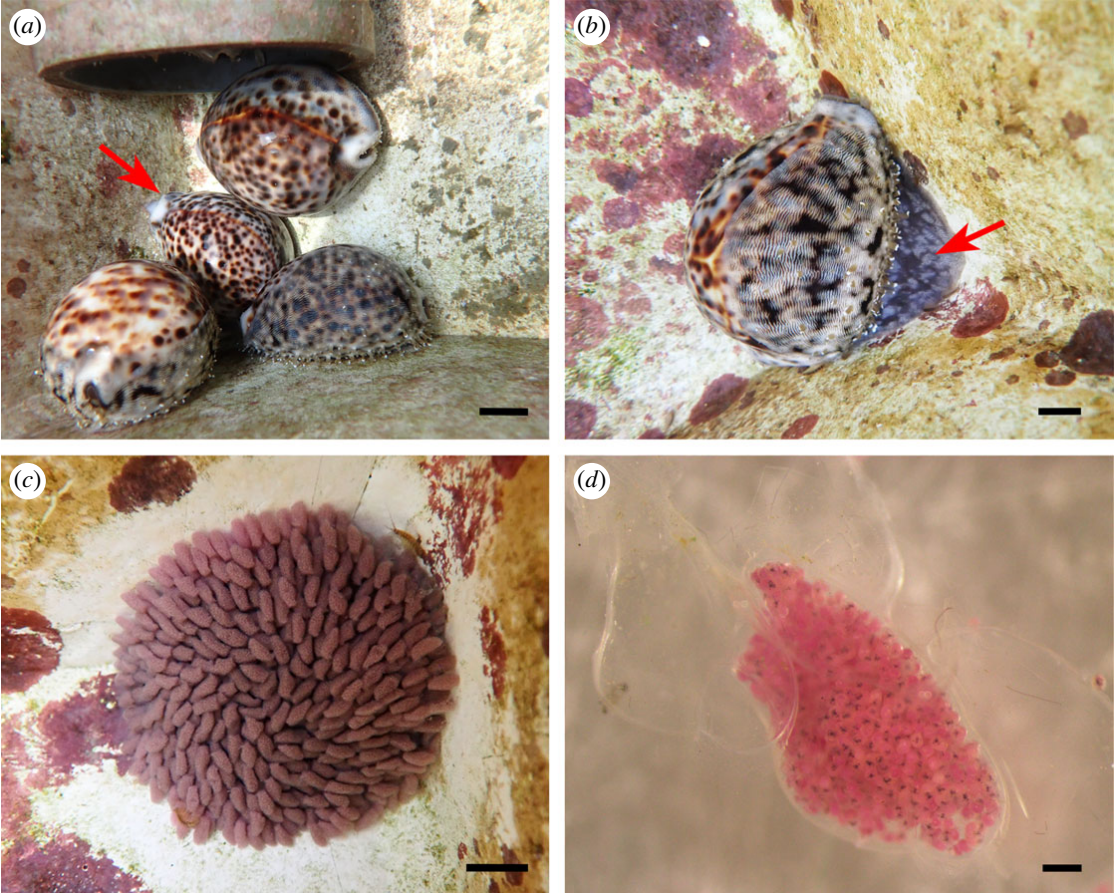
*Cypraea tigris*. (*a*) Clumping of cowries around a brooding cowrie (arrow); (*b*) brooding cowrie with a bulged foot (arrow); (*c*) exposed dark purple-red egg mass at 9 days post-spawning; (*d*) egg capsules containing developing veligers with surrounding empty gelatinous egg capsules. Scale bars: (*a*) 2 cm; (*b*) 1 cm; (*c*) 10 mm; (*d*) 500 µm.

Between March and September 2022, eight female *C*. *tigris* broodstock cowries collectively produced 20 egg masses, after being reared in captivity for approximately seven months (table 2). Multiple egg mass production was observed among five individuals, where cowries each laid between two to five egg masses consecutively with time intervals between 3 and 14 weeks (table 2). The brooder size ranged from 6.31 to 9.84 cm (mean: 7.56 cm ± s.d. 1.13 cm) in shell length and 86.60 to 268.38 g (mean: 142.71 g ± s.d. 63.74 g) in wet weight. An early indication of the presence of brooding *C*. *tigris* females was the clumping of cowries in groups of up to four individuals around a single brooding female during the egg laying and brooding period, and eventually dispersing before hatching occurs (figure 2*a*). Each clump could composed of both male and female cowries.

**Table 2.** Overview of brooding, spawning, egg mass and capsules for *C. tigris* and *M. arabica* from published studies and this study. Abbreviations: *L* = length; *W* = width; *N* = number of egg masses sampled.

	*Cypraea tigris*			*Mauritia arabica*	
study site	Xisha Islands	India	Singapore	Hainan Island	Singapore
spawning period	May 1957	October 2014	March – September 2022	April 1955, June 1957	March – September 2022
number of broodstock	—	6	14	—	7
number of brooders	1	1	8	3	3
number of spawning batches	1	1	20	3	6
estimated brooding period (days)	—	5	7–17	—	7–10
size of egg mass (*L* × *W* mm); *N*	68 × 60	—	30–70 × 25–50; *N* = 16	40 × 33	40–45 × 35–40; *N* = 5
size of capsules (*L* × *W* mm); *N*	4.5–5 × 2.6–2.8	2.55×1.44	3.76 (± s.d. 0.3)×1.83 (± s.d. 0.2); *N* = 15	2.5–3 × 1.3–1.6	2.57 (± s.d. 0.3) × 1.53 (± s.d. 0.2); *N* = 5
number of larvae per egg capsule; *N*	1000	686; *N* = 30^a^	737 (± s.d. 107); *N* = 15	520	459 (± s.d. 63); *N* = 5
reference	[28]	[33]	present study [36]	[28]	present study [36]

^a^number of capsules sampled.

In *C*. *tigris*, each egg mass was fully covered by the brooder's foot throughout the brooding period with limited exposure of egg capsules to the surroundings (figure 2*b*). Brooders exhibited very protective behaviour as they were difficult to pry away from their egg masses. Egg masses of *C*. *tigris* were usually circular and exhibited a purple-red colour when first laid that darkened over the brooding period (figure 2*c*). The capsules were mostly pyriform with gelatinous membranes (figure 2*d*). Capsules were arranged in layers, where the first layer was attached firmly to the substratum via a basal plate and subsequent layers glued to the underlying ones, making up to five layers in the entire mass. The number of larvae in each egg capsule varied between and within batches, ranging from 350 to 950 individuals per capsule (table 2). The brooding period for *C*. *tigris* lasted between 7 and 17 days, although the majority of the batches hatched within 11–13 days. Hatching usually took place over 1–3 days, with peak hatching (i.e. releasing 90% of total larvae) occurring overnight on the final day. Early embryogenesis was not observed in *C*. *tigris* due to the difficulty in obtaining egg capsules from protective brooders for observations.

#### 3.1.2. Larval and shell development

**Figure 3.**
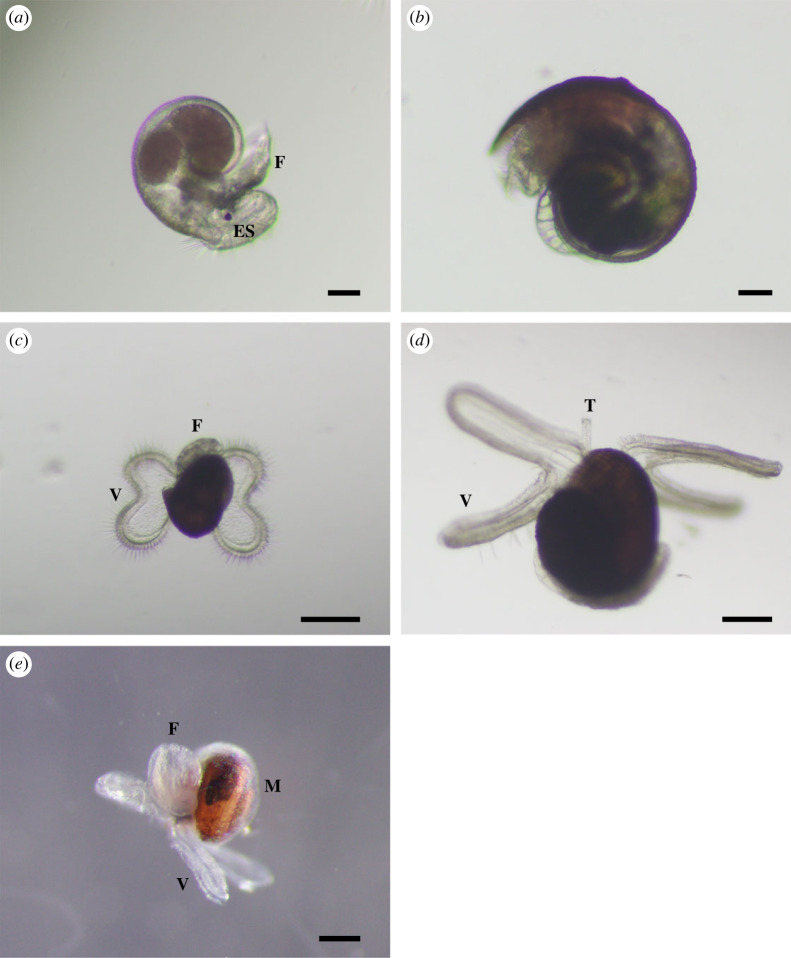
Morphology of *C. tigris* larva. (*a*) Newly hatched 0 dph larva; (*b*) 4 dph larva; (*c*) 8 dph larva with dividing velar lobes; (*d*) 20 dph larva with four velar lobes and left tentacle; (*e*) 31 dph larva with mantle. ES: eyespot; F: foot; M: mantle; T: tentacle; V: velum. Scale bars: (*a*,*b*) 50 µm; (*c*–*e*) 200 µm.

**Figure 4.**
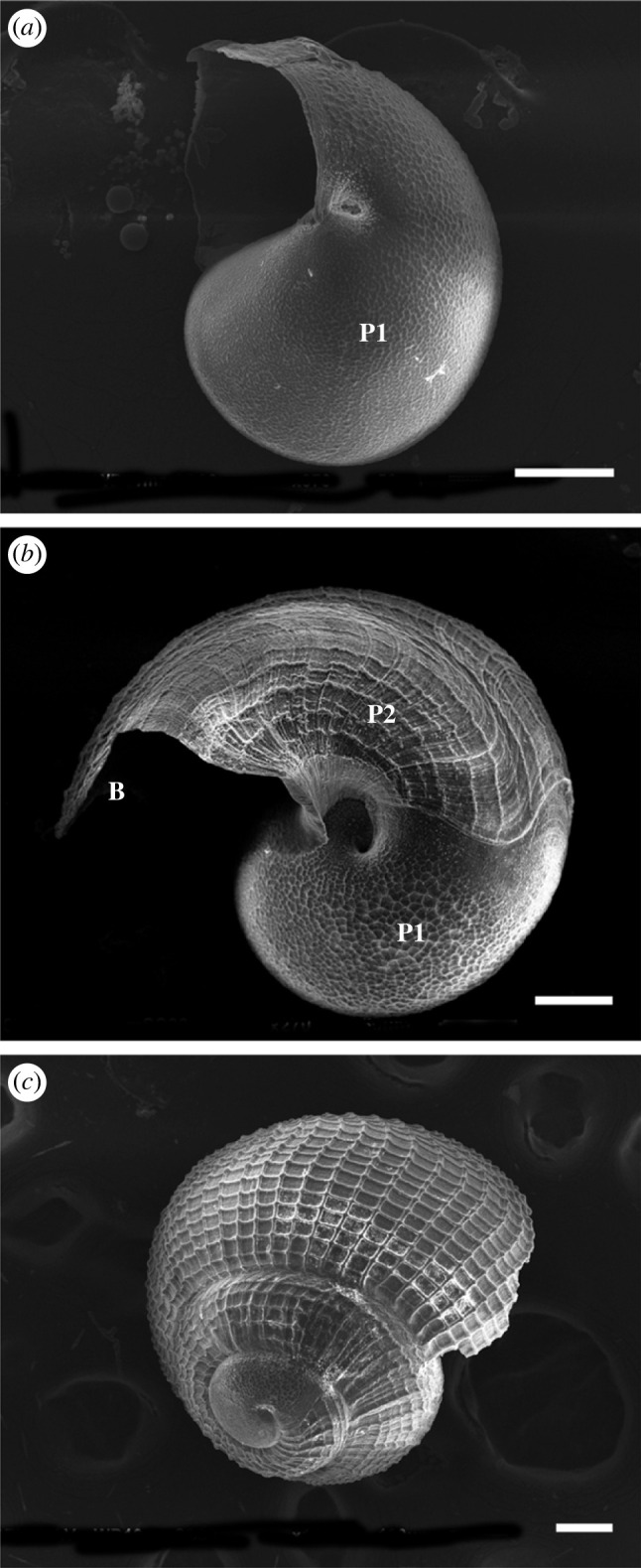
SEM images of *C. tigris* larval shells. (*a*) 0 dph protoconch I shell; (*b*) 4 dph protoconch II shell; (*c*) 26 dph shell with pronounced growth lines. B: apertural beak; P1: protoconch I; P2: protoconch II. Scale bars: (*a,b*) 50 µm; (*c*) 100 µm.

Protoconch I larvae (or veligers) of *C*. *tigris* were released from egg capsules during hatching, with a size range of 200–242 µm in shell length. Shells were largely translucent with a tinge of purple-red at the start of the first whorl. Newly hatched larvae possessed a bi-lobed velum, a pair of eyespots, along with a rudimentary foot and operculum (figure 3*a*). Protoconch I larvae had yet to complete the first whorl of their shells. Rapid shell growth was observed during the first 4 days post-hatching, where shell length increased up to approximately 300 µm (figure 8), along with the completion of first whorl by day 4, and increased opaqueness into a reddish-brown shell (figure 3*b*). The left tentacle (figure 3*d*) was first observed as early as 7 dph, while the velum expanded and elongated into four arms that were distinctly bifurcated by 14 dph. Under SEM, the surfaces of protoconch I were pitted, giving a cratered appearance (figure 4*a*), while protoconch II had spiral and axial ridges running across up to the aperture margin, with a visible line demarcating the boundary between protoconch I and protoconch II (figure 4*b*). A distinct apertural beak was also observed. By 26 dph, the shell had 2.5 whorls and the pronounced ridges led to rectangular grids covering protoconch II (figure 4*c*). The mantle was first observed around 30 dph (figure 3*e*) as a thin translucent tissue, which developed into a thicker and opaque mantle fully enveloping the shell by 33 dph. The largest shells observed had formed just over three whorls by 35 dph.

In general, greater than 80% of *C*. *tigris* larvae had survived in the first 4 days post-hatching, but a significant bottleneck was observed around 5–6 dph where greater than 50% of larvae died (figure 9). High rates of mortalities persisted up to 14 dph. A few batches of *C*. *tigris* larvae appeared to be infected by ciliates (*Vorticella* sp.) and algal fouling of larval shells was common. The longest surviving culture of *C. tigris* larvae in this study was 37 dph, although development appeared to have stagnated towards the end of culture and the shell remained in discoid form. No *C*. *tigris* larvae successfully settled during the culture period.

### 3.2. *Mauritia arabica* (Arabian cowrie)

#### 3.2.1. Brooding and spawning

**Figure 5.**
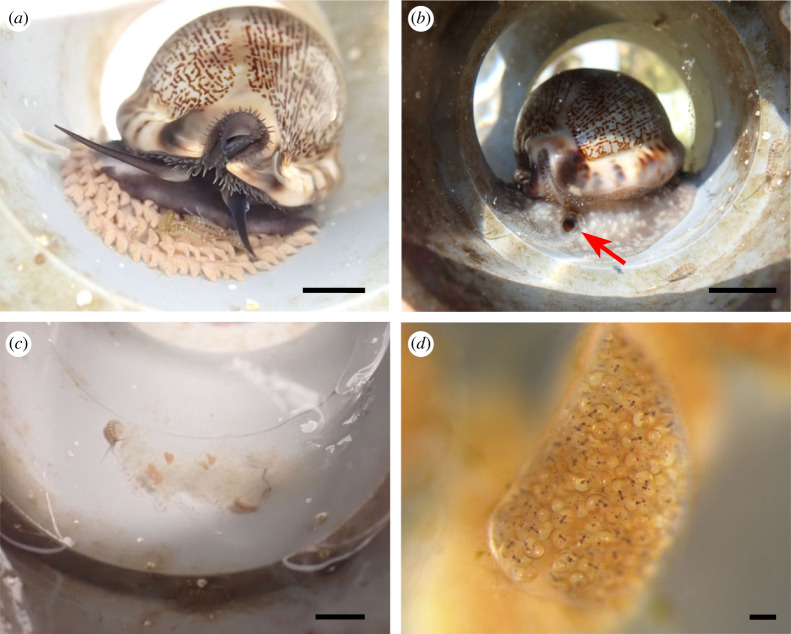
*Mauritia arabica*. (*a*) Brooding cowrie seated over egg mass; (*b*) brooding cowrie with a hole (arrow) in bulged foot allowing glimpses of egg mass; (*c*) remnants of translucent empty egg capsules post-hatching; (*d*) an intact egg capsule containing veligers. Scale bars: (*a*–*c*) 10 mm; (*d*) 200 µm.

Between March and September 2022, three female *M*. *arabica* broodstock cowries collectively produced six egg masses, after being reared in captivity for approximately five months (table 2). Two individuals were observed to produce multiple egg masses within a span of one–four months. Brooder size ranged from 6.0 to 6.5 cm (mean: 6.23 cm ± s.d. 0.20 cm) in shell length and 48.6–64.26 g (mean: 56.62 g ± s.d. 6.40 g) in wet weight. Brooding *M*. *arabica* cowries were found singly with their bulging foot shielding the egg mass (figure 5*a,b*). Newly laid egg masses of *M*. *arabica* exhibited cream coloration, which subsequently turned orange-pink around 3 days post-spawning (dps) (figure 5*d*). The number of larvae in each egg capsule ranged from 350 to 550. Egg capsules were transparent and gelatinous, with up to five layers of egg capsules. Hatching took place over the course of 1–3 days, after a brooding period of 7–10 days.

Exposed egg capsules of *M*. *arabica* were collected early for observations on embryogenesis. The fertilized eggs had developed into two- and four-celled embryos within 2–3 h. Trochophores were observed at 3 dps, before developing into shelled veligers at 5 dps. Within the capsules, the veligers were largely inactive but showed greater movement with distinct velum activity by 7 dps. At the end of the brooding period, the healthy larvae broke out from the capsules naturally.

#### 3.2.2. Larval and shell development

**Figure 6.**
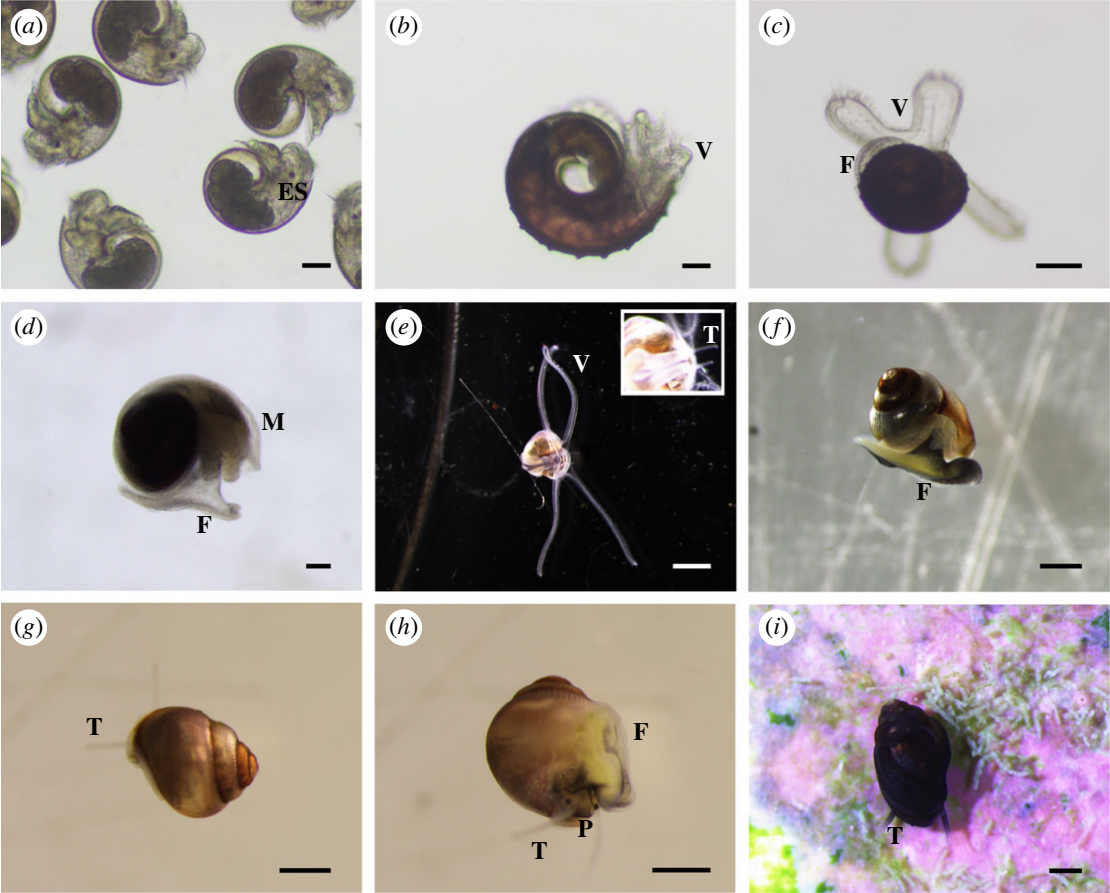
Morphology of *M. arabica* larva and juvenile. (*a*) Newly hatched 0 dph larvae; (*b*) 4 dph larva; (*c*) 14 dph larva with dividing velar lobes; (*d*) 26 dph larva with a well-developed foot and mantle; (*e*) 33 dph larva with elongated velar lobes and two tentacles; (*f*) 65 dph larva with elongated shell; (*g*) newly settled juvenile; (*h*) lateral view of newly settled juvenile; (*i*) juvenile at bulla stage. ES: eyespot; F: foot; M: mantle; P: proboscis; T: tentacle; V: velum. Scale bars: (*a*,*b*) 50 µm; (*c*,*d*) 200 µm; (*f*–*i*) 500 µm; (*e*) 1 mm.

The newly hatched *M. arabica* veligers were between 162 and 205 µm in size. The larvae had a bi-lobed velum, one pair of eyespots, a foot and an operculum (figure 6*a*). Shells had slightly greater than 0.5 whorl and were translucent. The left tentacle was first observed at 9 dph and the mantle started to appear at 14 dph as a thin translucent tissue, which could fully envelop the shell by 16 dph. The bi-lobed velum had also developed into four distinct velar arms around 14 dph (figure 6*c*), significantly elongating around 19 dph to more than twice the shell length. At 21 dph, the right tentacle started to develop but equal length tentacles were only observed around 35 dph (figure 6*e*), suggesting asynchronous development of these organs. By 30 dph, the foot had significantly enlarged, and the larva was still relatively mobile although its foot appears to lack the strength to move the shell considerably (figure 6*d*). Black pigmentation was also observed in the foot.

**Figure 7.**
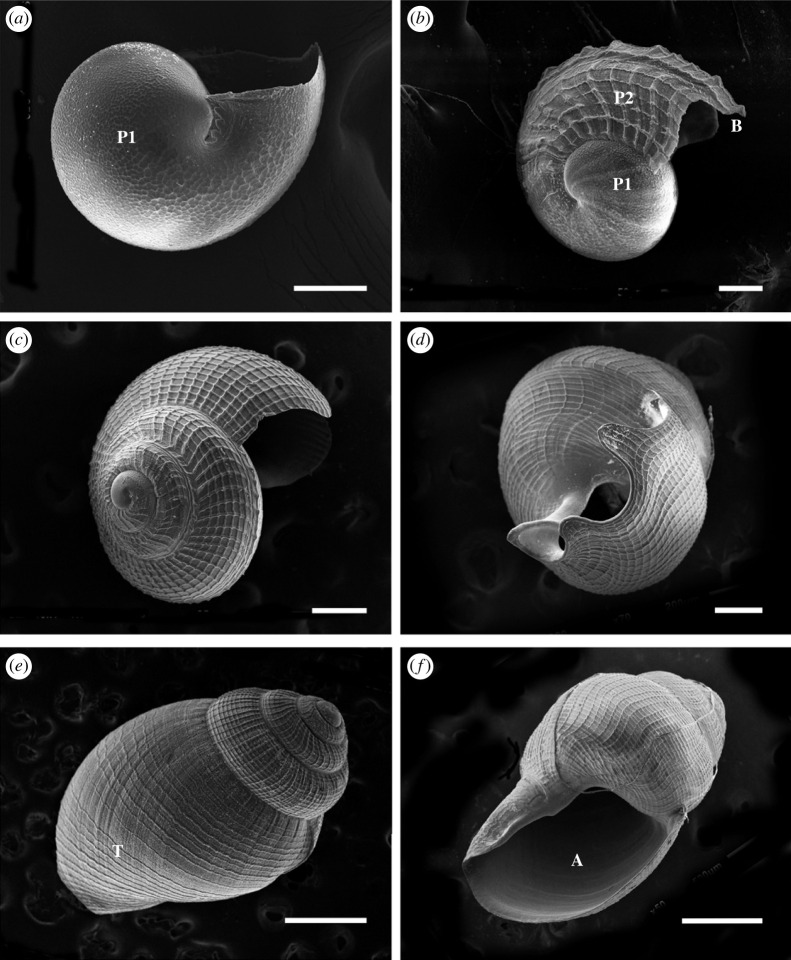
SEM images of *M. arabica* larval shells (*a*) 0 dph protoconch I shell; (*b*) 4 dph protoconch II shell; (*c*) 30 dph shell with 3.5 whorls; (*d*) 56 dph shell with notched basal lip; (*e*) dorsal view of juvenile; (*f*) ventral view of juvenile. A: aperture; B: apertural beak; P1: protoconch I; P2: protoconch II; T: teleoconch. Scale bars: (*a*,*b*) 50 µm; (*c*,*d*) 200 µm; (*e*,*f*) 500 µm.

Based on observations in this study, early indications of the larval competency for settlement were reduced swimming activity where the velum was no longer extended as frequently, followed by increased benthic activity on the bottom. Crawling was observed around 60 dph and juvenile metamorphosis was first noted at 70 dph, where the latter consisted of losing the velar lobes, developing a proboscis, and the loss of the operculum (figure 6*g*,*h*). Newly settled juveniles were also observed to graze on tiles encrusted with algae and diatoms. The shells further developed by elongation and narrowing of aperture to reach an olivoform shape (also known as bulla stage) (figures 6*i* and 7*e*,*f*), and their shells, mantles and tentacles darkened significantly.

Protoconch I shell was pitted (figure 7*a*) while the protoconch II shell was covered with spiral and axial ridges, forming rectangular grids (figure 7*b*). At 3–4 dph, visibly raised ridges could be seen along the apertural beak (figures 6*b* and 7*b*), appearing as triangular bumps, and shells were less transparent. The second whorl developed around 14 dph, and by 30 dph, most larvae had started to develop the third whorl (figure 7*c*). The fourth whorl expanded more rapidly than the previous whorls, giving the shell a more ovate shape (figure 6*f*). Late-stage larva had a sinusigera aperture and the basal lip developed two rounded notches (figure 7*d*).

**Figure 8.**
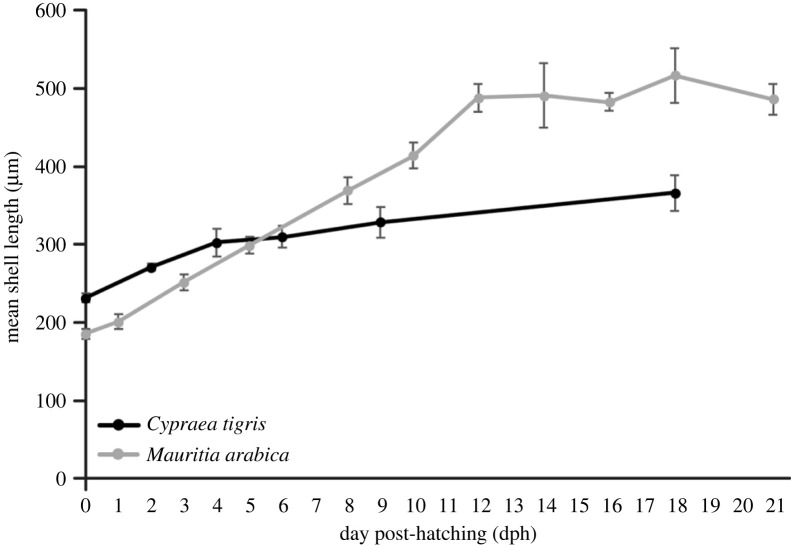
Mean shell length changes of early-stage *C*. *tigris* (*N* = 1) and *M*. *arabica* (*N* = 1) larvae for up to 21 dph; *N* = number of larval batches sampled.

The initial shell growth was almost linear, with shell lengths increasing to an average of 500 µm within the first two weeks (figure 8) but reaching a plateau thereafter until 30 dph. Survival rates throughout the culture period were generally high in *M*. *arabica*, with cumulative mortality at 80% by 30 dph. Observations on late-stage larvae showed no further growth or development, until the larvae either settled or eventually died.

## 4. Discussion

### 4.1. Brooding and spawning

Two tropical species of cowries, *C*. *tigris* and *M*. *arabica*, were observed to spawn in captivity. Notably, mating cowries (i.e. copulating pairs) for both species were not positively identified throughout this observation period. The brooding females of both cowrie species in this study had mostly likely mated *ex situ* considering the long captivity duration; although the female cowries can store sperm for fertilization and deposit eggs much later [37]. While clumps of two–four cowries were observed for *C*. *tigris* (but not for *M*. *arabica*), they usually consisted of both male and female individuals with no visible signs of copulation behaviour. Instead, these clumps usually signify the presence of an egg mass and a brooding female that has either already spawned or is spawning. Such clumping and gregarious behaviour have been observed in other cowrie species such as the *Ovula ovum*, where pair formations are common and a male cowrie next to a brooding female could be a form of mate guarding and protection of eggs [37,38]. *Monetaria caputserpentis* also displays gregarious behaviour during brooding, where males and females coexist with the brooding female [39], while Appeldoorn [40] proposed that the aggregation of cowries could be influenced by the release of brooder's pheromones.

Apart from clumping, the brooding females of *C*. *tigris* and *M*. *arabica* were also typically identified by its bulged foot and sessile state for an extended period of time. Another behavioural observation of brooding females was the clean, algae-free surfaces at the spawning sites, which suggested that they cleaned the site before depositing egg capsules, probably for better attachment of capsules to the surface. In only *M*. *arabica*, a small hole in the posterior end of its foot could be seen during brooding, possibly for aerating the eggs (figure 5*b*). When a brooder was removed from the egg mass during the brooding period, it would slowly crawl back to its egg mass, which concurs with previous studies [12,31]; although this behaviour was not consistent across brooding individuals and between species in this study. Three *C*. *tigris* brooders were removed from their egg mass on separate occasions during the laying of egg capsules, but they responded differently to the disturbances. The first one was able to return to its egg mass and continued laying egg capsules, while the second and third cowries discarded their egg masses, either via consumption or no resumption of egg laying. By contrast, disturbance to brooder *M*. *arabica* had largely led to the permanent abandonment and/or consumption of egg masses, despite our observations of robust egg capsules and developing larvae observed. On two occasions, the egg masses were consumed during brooding period despite being left undisturbed. Other species such as *Lyncina carneola* [27] and *Erronea errones* [23] have reportedly abandoned their eggs after being disturbed, although the reasons were not elaborated. Even though the consumption of egg capsules during brooding is considered unusual, Renaud [22] suggested that cowries may eat the capsules if the embryos were non-viable or if the egg mass had been disturbed. Between the two species in our study, *M. arabica* had a higher incidence of egg mass abandonment (four out of six egg masses) compared with *C. tigris* (four of 20 egg masses), suggesting that *M*. *arabica* may be more sensitive to handling and removing of brooders for observations throughout brooding.

The information on the spawning and larval development of Cypraeidae larvae remains limited for most species, with the exception of a single study on *C*. *tigris* by Jagadis *et al*. [33]. When comparing the results from this study with Jagadis *et al*. [33], there are several notable differences in the egg mass characteristics and larval morphology of *C*. *tigris*. First, Jagadis *et al*. [33] noted that their *C. tigris* egg mass was pale grey, turning to dark grey after 4 dps, while the egg masses here were purple-red throughout the brooding period. Both colours have also been documented from *C. tigris* in the wild: purple-red in Tanzania [41] and greyish-brown in Xisha Islands [28], implying that the environmental conditions could influence the egg mass colour or that the egg masses could represent different subspecies. Second, Jagadis *et al*. [33] reported a brooding period of 5 days that produced hatched veligers of sizes 550–590 µm, where *C*. *tigris* in this study brooded over the egg mass for up to 17 days (with the shortest brooding period at 7 days) and hatched veligers were half the size. Another study by Qi & Ma [28] reported their *C*. *tigris* larval sizes at 210–240 µm, which complements with current observations here. Additional observations of *C*. *tigris* spawnings from different geographical locations are needed before these morphological differences can be explained.

Results for *M*. *arabica* spawning and larval development are reported herein for the first time. In general, the spawning success of their egg masses under laboratory conditions varied due to high egg mass abandonment rates, which resulted in batches that consisted of abnormal embryos and trochophores or degenerated embryos with few capsules containing protoconch I veligers. Capsules with non-viable embryos remained cream-coloured while those with normal developing embryos would turn orange-pink. Several had exhibited bright pink coloration, which is a sign of decay or infection. It appears that parental care for the egg masses is critical for *M*. *arabica* to ensure a higher chance of producing viable larvae. Despite the lack of or no brood care, some *M*. *arabica* egg masses contained embryos that developed normally and eventually hatched naturally, indicating that normal larvae development may continue for abandoned egg masses. Previous studies of other cowrie species have also shown mixed outcomes for abandoned egg masses or egg masses without brood care. In the absence of a brooder, Pang [23] found that freshly laid egg masses did not develop further and degraded after a week whereas Ostergaarg [27] managed to successfully hatch and rear several species of larvae.

The first signs of pre-hatching are the increased swimming activity of protoconch I veligers with small velar lobes within the capsules. Some studies have found that the female brooder nips at the capsules to break them during hatching, allowing the larvae to escape quickly [42]. In this study, we did not observe any brooders of *C*. *tigris* and *M*. *arabica* actively nipping at the capsules to release the larvae. Instead, the larvae were found to break out of the capsules on their own via an off-centre escape aperture at the top of the capsule by bumping against the walls and exerting pressure against the membrane. Newly hatched *C*. *tigris* and *M*. *arabica* larvae are also positively phototactic, which coincides with prior observations of cowries and other gastropod species [32,43]. After hatching completes, the egg mass site is usually bare or has a few empty egg capsules still attached to the surface. It is also common to find remnants of empty capsules scattered near the original egg mass. Tanaka [32] mentioned that the brooders would consume the empty egg capsules, probably as a form of nutrition. This would be highly necessary considering that the brooders would have eaten little during the brooding period, besides scrapping algae within its immediate vicinity.

### 4.2. Larval and shell development

**Figure 9.**
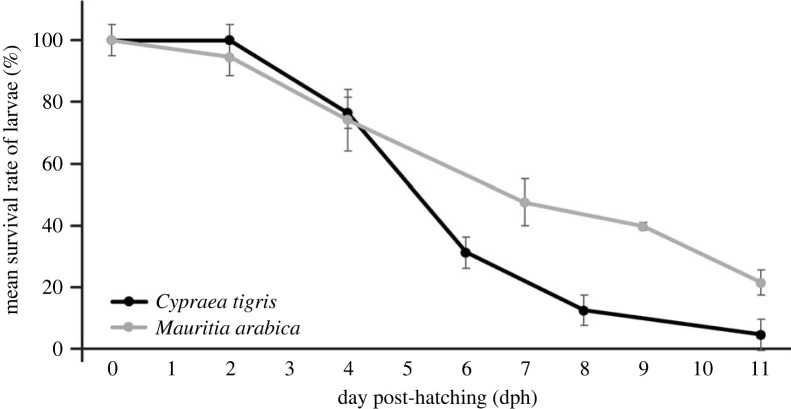
Average survival rates of *C*. *tigris* (*N* = 5) and *M*. *arabica* (*N* = 4) larvae for up to 11 dph; *N* = number of larval batches sampled.

In the laboratory cultures, *M. arabica* larvae appear to be more robust, catching up to and surpassing *C. tigris* larvae in terms of shell length within the first week post-hatching (figure 8). Generally, the development of *M*. *arabica* was faster with higher survival rates (figure 9), and the larvae can be reared under laboratory conditions for more than four months. Although the metamorphosis and settlement process for cowries are still not well understood, our study had successfully reared *M. arabica* larvae for more than four months with observations of *M. arabica* larval metamorphosis and settlement. Unfortunately, the longest surviving *C. tigris* larvae lasted up to just 37 days, and their numbers were few and stagnated development transpired. The first *M. arabica* juvenile was observed at 70 dph, but mortality occurred soon after. Larval settlement can be energetically expensive for marine invertebrates [44,45], and the lack of adequate nutrition thereafter could have contributed to the loss of individuals. The remaining *M. arabica* individuals metamorphosed between 80 and 100 dph, chiefly characterized by the loss of velum, appearance of proboscis, and were actively crawling and grazing upon the introduced tiles with encrusting algae. Over time, these juveniles developed to the bulla stage where the shells darkened and elongated, with a narrower aperture.

The metamorphosis of *M*. *arabica* larvae occurred spontaneously in the absence of a settlement cue for our study. Despite the presence of competent larvae, the majority of them did not undergo metamorphosis even after more than four months, suggesting that a specific settlement cue is required, but not examined in this study due to low sample size. Some gastropod species are known to have competent larvae with the capacity to enter a delay period or maintain a non-growing state for extended periods, and metamorphose only in the presence of a specific cue [46]. Settlement on dead adult conspecific shells is induced in the muricid *Concholepas concholepas* larvae in the presence of barnacles on these shells [47], while competent *Strombus pugilis* larvae are able to settle naturally after 29 days with no specific cues needed [48]. Among the other molluscs, the larvae of sea slug *Aplysia juliana* cultured in the laboratory are competent at 30 dph, but can survive for as long as 300 dph and will only metamorphose in the presence of *Ulva* sp. [49]. Further work is needed to understand settlement cues in cowrie larvae as a prolonged planktonic phase may negatively impact larval condition and survival in the long term, as observed by the high mortality rates of older larvae.

Ornamentation of larval shells can be used as a taxonomic character to identify and distinguish gastropod species. This is the first study documenting the larval shell morphology of two tropical cowrie species. Broadly, the *M. arabica* shells are smaller in size than *C. tigris* when first hatched, and the early *M. arabica* larvae have prominently raised ridges along the apertural beak, which eventually erodes, but these ridges are absent in *C*. *tigris*. On the other hand, the larval shells of both Cypraeidae species have several similarities. Their protoconch I shell surfaces are pitted with shallow depressions, and like all other Cypraeidae species, protoconch II shells have rectangular grids formed by spiral and axial grids [50]. The granular sculptures of *C. tigris* and *M. arabica* protoconch I are also found in relative species of Ovulidae, *Simnia barbarensis* and *S. aequalis*, all of which are from the superfamily Cypraeoidea. To distinguish between the families Cypraeidae and Ovulidae, the ornamentation of whorls in Ovulidae shells possess oblique rectangular grids due to prosocline and opisthocline ribs compared with the orthocline ribs in Cypraeidae [50]. Within the subclass Caenogastropoda, other families including Triphoridae and Turridae are also known to have similar grids, while other species such as *Turrritella cingulata* (family Turritellidae) and *Cerithiopsis* sp. (family Cerithiopsidae) only have fine axial lines, and *Janthina* sp. (family Janthinidae) is covered with axial reliefs interspersed by small notches [51,52].

## 5. Conclusion

The spawning and brooding behaviour of the *C. tigris* and *M. arabica* in captivity are reported and the larval development of both species are fully described for the first time. The first successful metamorphosis of *M. arabica* into juvenile and development to bulla stage is also documented. Additional work needs to be done to determine if and what specific settlement cues would induce metamorphosis and settlement in cowrie larvae. Considering the relatively long planktonic phase, the *ex situ* culture conditions also need to be refined, particularly for *C. tigris*, in order to improve larval development and survivorship. The findings from this study now provide the basis to understand early life history of cowries that can be used to further reproduction studies, as well as to use the ecological information for informing the management of the wild populations with respect to conservation and ensuring sustainable aquarium trade.

## Data Availability

Data available from the Dryad Digital Repository: https://doi.org/10.5061/dryad.02v6wwq6h [36].

## References

[1] Moretzsohn F. 2014 Cypraeidae: how well-inventoried is the best-known seashell family? Am. Malacol. Bullet. **32**, 278-289. (10.4003/006.032.0219)

[2] Passamonti M. 2015 The family Cypraeidae (Gastropoda Cypraeoidea) an unexpected case of neglected animals. Biodivers. J. **6**, 449-466.

[3] Poulsen AL. 1995 Coral reef gastropods – a sustainable resource? Pacific Conserv. Biol. **2**, 142-145. (10.1071/PC960142)

[4] Ardines R, Mecha NJM, Dolorosa R. 2020 Commonly gleaned macro-benthic invertebrates in a small offshore island of Cawili, Cagayancillo, Palawan, Philippines. The Palawan Scientist **12**, 102-125.

[5] Jagadis I. 2000 Shell industries of Rameswaram Islands. Souvenir 2000, pp. 50-52. Tamil Nadu, India: Central Marine Fisheries Research Institute.

[6] Babu A, Venkatesan V, Rajagopal S. 2011 Contribution to the knowledge of ornamental molluscs of Parangipettai, southeast coast of India. Adv. Appl. Sci. Res. **2**, 290-296.

[7] Yang B. 2011 The rise and fall of cowrie shells: the Asian story. J. World Hist. **22**, 1-25. (10.1353/jwh.2011.0011)

[8] Padhi N. 2021 Kingdom of seashells: explotation and culture of major commercial species. Int. J. Aquacult. Fishery Sci. **7**, 001-004. (10.17352/2455-8400.000065)

[9] Kay A. 1960 Generic revision of the Cypraeinae. J. Mollusc. Stud. **33**, 278-287. (10.1093/oxfordjournals.mollus.a064832)

[10] Irie T, Iwasa Y. 2003 Optimal growth model for the latitudinal cline of shell morphology in cowries (genus *Cypraea*). Evol. Ecol. Res. **5**, 1133-1149.

[11] Irie T. 2006 Geographical variation of shell morphology in *Cypraea annulus* (Gastropoda: Cypraeidae). J. Mollusc. Stud. **72**, 31-38. (10.1093/mollus/eyi043)

[12] Wilson B. 1985 Direct development in southern Australian cowries (Gastropoda: Cypraeidae). Mar. Freshw. Res. **36**, 267-280. (10.1071/MF9850267)

[13] Villamor S, Yamamoto T. 2015 Population characteristics of *Monetaria annulus* (Linnaeus, 1758) (Gastropoda: Cypraeidae) from temperate to tropical areas. Aquacult. Sci. **63**, 273-282. (10.11233/aquaculturesci.63.273)

[14] Brock RE. 1979 A statistical study of *Cypraea tigris* in the Central Pacific. The Veliger **22**, 166-170.

[15] Chou L, Tan K. 2008 Corals, worms and molluscs. In The Singapore red data book: threatened plants and animals of Singapore (eds GWH Davidson, PKL Ng, HC Ho), pp. 39-61. Singapore: The Nature Society.

[16] Poutiers JM. 1998 Gastropods FAO species identification guide for fishery purposes. In *The living marine resources of the Western Central Pacific. Volume 1**:* Seaweeds, Corals, Bivalves Gastropods. FAO.

[17] Vicente J, Osberg A, Marty MJ, Rice K, Toonen RJ. 2020 Influence of palatability on the feeding preferences of the endemic Hawaiian tiger cowrie for indigenous and introduced sponges. Mar. Ecol. Prog. Ser. **647**, 109-122. (10.3354/meps13418)

[18] Reese DS. 1991 The trade of Indo-Pacific shells into the Mediterranean Basin and Europe. Oxford J. Archaeol. **10**, 159-196. (10.1111/j.1468-0092.1991.tb00012.x)

[19] Raj KD, Mathews G, Kumar PD. 2019 Tiger cowrie *Cypraea tigris* feeds on coral-competing sponge *Rhabdastrella globostellata* in an Acropora dominated reef of Gulf of Mannar, India. Mar. Freshw. Behav. Physiol. **52**, 101-105. (10.1080/10236244.2019.1637701)

[20] Schilder FA. 1961 Another statistical study in size of cowries. The Veliger **4**, 107-112.

[21] Burgess CM. 1985 Cowries of the world. Cape Town, South Africa: Seacomber Publications.

[22] Renaud ML. 1971 Aspects of the biology and ecology of *Cypraea moneta* at Eniwetok Atoll, Marshall Islands. Doctoral dissertation, University of Hawaii, Honolulu, HI.

[23] Pang JSY. 1969 Some studies on the biology of *Cypraea errones* Linnaeus. Honors thesis, National Univerity of Singapore, Singapore.

[24] D'Asaro CN. 1970 Egg capsules of prosobranch mollusks from south Florida and the Bahamas and notes on spawning in the laboratory. Bull. Mar. Sci. **20**, 414-440.

[25] Chaparro O, Oyarzun R, Vergara A, Thompson R. 1999 Energy investment in nurse eggs and egg capsules in *Crepidula dilatata* Lamarck (Gastropoda, Calyptraeidae) and its influence on the hatching size of the juvenile. J. Exp. Mar. Biol. Ecol. **232**, 261-274. (10.1016/S0022-0981(98)00115-4)

[26] Vafiadis P, Burn R. 2020 Internal embryonic brooding and development in the southern Australian micro-snail *Tricolia rosea* (Angas, 1867) (Vetigastropoda: Phasianellidae: Tricoliinae). Mollusc. Res. **40**, 60-76. (10.1080/13235818.2019.1672251)

[27] Ostergaarg JM. 1950 Spawning and development of some Hawaiian marine gastropods. Pacific Sci. **4**, 75-115.

[28] Qi Z, Ma X. 1988 Study on the egg masses of 12 species of Cypraeidae. Chin. J. Oceanol. **6**, 171-178. (10.1007/BF02847836)

[29] Richards Z et al. 2016 Marine biodiversity in temperate Western Australia: multi-taxon surveys of Minden and Roe Reefs. Diversity **8**, 7. (10.3390/d8020007)

[30] Doneddu M. 1999 Some notes about the range, habitat and ecology of the Mediterranean species of Cypraeidae. The Festivus **31**, 87-91.

[31] Natarajan A. 1954 On the breeding habits of the cowry *Erronea errones* (Linne). Curr. Sci. **23**, 225-226.

[32] Tanaka Y. 1980 Spawning and development of the cowry, *Cypraea (Ponda) vitellus* Linnaeus. Venus **39**, 117-122.

[33] Jagadis I, Kavitha M, Padmanathan J, Maharshi A, Varadarajakumar A. 2017 Lessons on broodstock maintenance, spawning, larval rearing and juvenile production of marine gastropods of ornamental value. Aquacult. Res. **48**, 2581-2592. (10.1111/are.13094)

[34] Tay TS, Gan BQ, Lee SCS, Lim CS, Tan KS, Teo SLM. 2018 Larval development of the invasive charru mussel, *Mytella strigata* (Bivalvia: Mytilidae). Invertebr. Reprod. Dev. **62**, 248-256. (10.1080/07924259.2018.1505671)

[35] Knight JB et al. 1960 Treatise on invertebrate paleontology, part 1, mollusca 1, pp. 185-1165. Lawrence, Kansas: Geological Society of America and University of Kansas.

[36] Tay TS, Lee HRS, Neo ML. 2023 Data from: Spawning and larval development of two tropical cowries (Gastropoda: Cypraeidae), *Cypraea tigris* and *Mauritia arabica* under laboratory conditions. *Dryad Digital Repository*. (10.5061/dryad.02v6wwq6h) PMC10090884 37063987

[37] Kawai K. 2010 Pair formation and reproductive behavior in the egg cowry *Ovula ovum* (Gastropoda: Ovulidae) in southern Kyushu, Japan. Venus: the Japanese J. Malacol. **69**, 49. (10.18941/venus.69.1-2_49)

[38] Kawai K. 2009 Shell growth, reproduction and mortality of *Ovula ovum* in southern Kyushu, Japan. J. Mollus. Stud. **75**, 35-40. (10.1093/mollus/eyn034)

[39] Osorio C, Brown D, Donoso L, Atan H. 1999 Aspects of the reproductive activity of *Cypraea caputdraconis* from Easter Island (Mollusca: Gastropoda: Cypraeidae). Pacific Sci. **53**, 15-23.

[40] Appeldoorn RS. 1995 Potential depensatory mechanisms operating on reproductive output in gonochoristic molluscs, with particular reference to strombid gastropods. In *ICES Marine Science Symp.,* *Mariehamn, Aland*, *pp.* 13-18. Copenhagen, Denmark: International Council for the Exploration of the Sea.

[41] Yaninek JS. 1978 A comparative survey of reef-associated gastropods at Maziwi Island, Tanzania. J. Afr. Natural Hist. Soc. Mus. **31**, 1-16.

[42] Katoh M. 1989 Life history of the golden ring cowry *Cypraea annulus* (Mollusca: Gastropoda) on Okinawa Island, Japan. Mar. Biol. **101**, 227-234. (10.1007/BF00391462)

[43] Fuchs HL, Solow AR, Mullineaux LS. 2010 Larval responses to turbulence and temperature in a tidal inlet: habitat selection by dispersing gastropods? J. Mar. Res. **68**, 153-188. (10.1357/002224010793079013)

[44] Botello G, Krug PJ. 2006 ‘Desperate larvae' revisited: age, energy and experience affect sensitivity to settlement cues in larvae of the gastropod *Alderia* sp. Mar. Ecol. Prog. Ser. **312**, 149-159. (10.3354/meps312149)

[45] He J, Wu Z, Chen L, Dai Q, Hao H, Su P, Ke C, Feng D. 2021 Adenosine triggers larval settlement and metamorphosis in the mussel *Mytilopsis sallei* through the ADK-AMPK-FoxO pathway. ACS Chem. Biol. **16**, 1390-1400. (10.1021/acschembio.1c00175) 34254778

[46] Scheltema RS. 1967 The relationship of temperature to the larval development of *Nassarius obsoletus* (Gastropoda). Biol. Bull. **132**, 253-265. (10.2307/1539893) 29332441

[47] Manríquez PH, Navarrete SA, Rosson A, Castilla JC. 2004 Settlement of the gastropod *Concholepas concholepas* on shells of conspecific adults. J. Mar. Biol. Assoc. UK **84**, 651-658. (10.1017/S0025315404009695h)

[48] Enriquez-Diaz M, Volland JM, Chavez-Villegas J, Aldana-Aranda D, Gros O. 2015 Development of the planktotrophic veligers and plantigrades of *Strombus pugilis* (Gastropoda). J. Mollus. Stud. **81**, 335-344. (10.1093/mollus/eyv011)

[49] Kempf SC. 1981 Long-lived larvae of the gastropod *Aplysia juliana*: do they disperse and metamorphose or just slowly fade away. Mar. Ecol. Prog. Ser. **6**, 61-65. (10.3354/meps006061)

[50] Bandel K, Riedel F, Weikert H. 1997 Planktonic gastropod larvae from the Red Sea: a synopsis. Ophelia **47**, 151-202. (10.1080/00785236.1997.10428670)

[51] Romero MS, Valdebenito EL. 2002 Larvas veliger de gastrópodos Prosobranchia provenientes de punta de Lobos, Cuarta Región, Chile. Revista chilena de historia natural **75**, 491-514. (10.4067/S0716-078X2002000300003)

[52] Hickman CS. 2004 The problem of similarity: analysis of repeated patterns of microsculpture on gastropod larval shells. Invertebr. Biol. **123**, 198-211. (10.1111/j.1744-7410.2004.tb00155.x)

